# Bevacizumab (Avastin) and Thermal Laser Combination Therapy for Peripapillary Choroidal Neovascular Membranes

**DOI:** 10.1155/2017/4802690

**Published:** 2017-03-26

**Authors:** Sean D. Adrean, Scott Grant, Siyang Chaili

**Affiliations:** ^1^Retina Consultants of Orange County, Fullerton, CA, USA; ^2^The George Washington University School of Medicine and Health Sciences, Washington, DC, USA

## Abstract

*Objective*. This is a retrospective interventional case series describing the results of 5 eyes from 5 patients with symptomatic peripapillary choroidal neovascularization (CNVM) receiving initial bevacizumab treatment followed by thermal laser and bevacizumab combination therapy. *Methods*. Patients received intravitreal bevacizumab injections until the lesions were well-defined. Thermal laser ablation was then administered and followed by an additional bevacizumab injection after one week. Visual outcomes, OCT changes, and rates of recurrence were recorded and analyzed. *Results*. Median visual outcomes improved from 20/50 to 20/30 (*p* = 0.0232). Median central macular thickness decreased from 347 *μ*m to 152 *μ*m (*p* = 0.0253). The mean visual improvement was 3 lines. An average of 3.8 bevacizumab injections per patient were given overall. Patients were followed for an average of 24 months, during which all eyes were absent for recurrence. *Conclusion*. Symptomatic peripapillary CNVM may be successfully managed with bevacizumab followed by a combination of thermal laser and bevacizumab without the need for frequent retreatment. The area requiring treatment may be better defined using bevacizumab, limiting the ablation of the healthy retina and improving treatment margins. With this treatment regimen, the patients experience improved visual outcomes and have a low rate of recurrence.

## 1. Introduction

Peripapillary or juxtapapillary choroidal neovascular membranes (CNVM) occur in patients due to a variety of conditions. These CNVM are most often associated with age-related macular degeneration (45%) or from idiopathic causes (39%) [1]. Peripapillary CNVM are also known to occur with other diseases including ocular histoplasmosis (OHS), angioid streaks, pars planitis, optic disc drusen, choroidal osteoma, and pattern dystrophies [1]. Peripapillary CNVM are predominately occult membranes and are often associated with retinal hemorrhage [2–4]. Some patients are asymptomatic and may be observed, while other patients present with decreased vision and metamorphopsia. Symptomatic patients often require treatment. Traditionally, these CNVM were ablated with thermal laser if 1.5 clock hours of retina temporal to the optic nerve was spared [1, 5–8]. Other treatment modalities have been utilized including surgical removal of large membranes associated with OHS [9] and photodynamic therapy [10, 11].

Anti-VEGF agents including intravitreal bevacizumab (Avastin) and ranibizumab are used to treat subfoveal CNVM associated with age-related macular degeneration [12, 13]. These agents are also being utilized to treat various other conditions associated with choroidal neovascularization, including peripapillary CNVM [14, 15]. There is also a rationale for combining various therapies for CNVM associated with subfoveal lesions [16] or peripapillary lesions [17]. Thus, in this case series, we examined the outcomes in peripapillary CNVM treated first with bevacizumab, followed by thermal laser and bevacizumab in combination. Visual outcomes were examined as well as the frequency of injections and stability of the CNVM after treatment.

## 2. Methods

This was a retrospective, noncomparative, interventional case series involving 5 eyes from 5 patients with peripapillary CNVM identified in clinical practice. Patients who had less than six months of follow-up, were asymptomatic, or underwent previous treatment were excluded from the study. All patients exhibited decreased vision and metamorphopsia. Patients had hemorrhage around the CNVM, exudates, and fluid involving the subfoveal region. Patients were initially evaluated with a complete ophthalmic examination and fluorescein angiogram (FA). Optical coherence tomography (OCT) was performed at each follow-up visit.

Following complete evaluation, treatment was initiated with 1.25 mg/0.05 ml intravitreal bevacizumab (Avastin, Genentech, San Francisco) under sterile conditions via pars plana injection. Patients returned for further evaluation at four- to six-week intervals. Complete ophthalmic and repeat OCT examinations were performed at each follow-up visit. Bevacizumab injections were repeated until the blood had resolved, the central macular thickness (CMT) was normalized, and the CNVM appeared stabilized clinically. A repeat FA was obtained to reexamine the lesion once the CNVM was smaller and well-defined.

Thermal laser (532 nanometer diode laser) was then applied to the CNVM, and 200 microns beyond the temporal side of the lesion until retinal whitening occurred. The optic nerve was not involved in the treatment. Treatment was administered according to the Macular Photocoagulation Study (MPS) guidelines [8]. Within one week of laser treatment, an additional bevacizumab injection was given. Patients were then evaluated for treatment success and safety by OCT at 1 month and carefully monitored for any signs of recurrence at increasing intervals of 6 weeks, 8 weeks, 10 weeks, and then every 3 months, for a minimum of 15 months.

Pre- and postoperative values for visual acuity and CMT were compared using the Wilcoxon rank-sum test. A *p* value less than 0.05 was considered statistically significant.

## 3. Results

**Figure 1:**
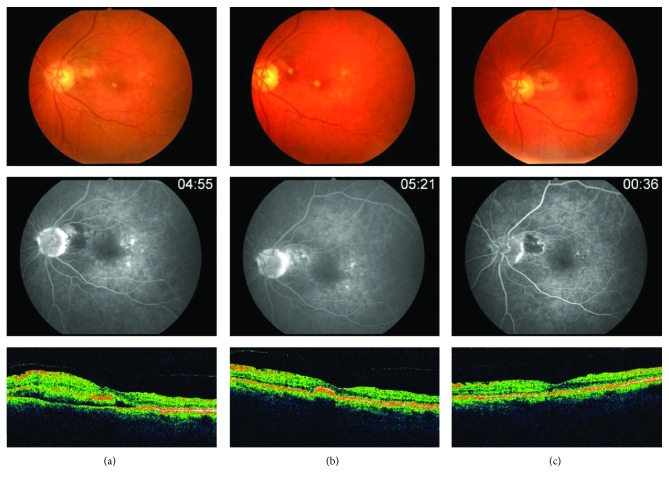
Retinal changes on fundoscopy, FA, and OCT throughout the treatment course. (a) At presentation, with fundus photos, FA, and OCT; (b) after Avastin treatments, prior to thermal ablation of the CNVM; and (c) after thermal laser and final Avastin treatment.

This study consisted of 3 females and 2 males with an average age of 74.8 years (range 56–84 years). All patients presented with occult membranes. Four patients were phakic, and 1 patient was pseudophakic. Evidence of AMD in both eyes with drusen and retinal pigment epithelial mottling were also observed in 4 patients. The average initial lesion size including hemorrhage and exudates covered approximately 2 disk areas (range 1–3 disc areas; see Figure 1). The mean number of clock hours that the CNVM surrounded the optic nerve was 3.8 (range 3–6). All lesions spared more than 1.5 clock hours of temporal retina.

The average follow-up period from the first diagnosis of juxtapapillary CNVM was 31 months (range 25–34 months). Each patient received an average of 2.8 bevacizumab injections (range 2–4 injections) prior to thermal laser treatment. An additional bevacizumab injection was given within one week. CNVM recurrence was absent for all patients over an average follow-up period of 24 months (range 21–28 months; see Table 1) upon completion of thermal laser and final bevacizumab treatments.

The median preoperative vision was 20/50 (range 20/40 to 20/80; see Table 1). Final median postoperative vision was 20/30, with visual acuity ranging from 20/20 to 20/40 (*p* = 0.0232). Visual improvement was observed for all patients (100%), and the average visual gain was +3.0 lines of vision. Two patients had 4 lines of visual improvement, 2 additional patients improved by 3 lines, and the final patient gained 1 line of vision. The median preoperative CMT foveal was 347 microns and decreased to a median of 152 microns (*p* = 0.0253) following intervention.

## 4. Discussion

This retrospective study demonstrates that combination therapy using bevacizumab and thermal laser for peripapillary CNVM improves vision and metamorphopsia, decreases CMT on OCT, and may limit CNVM recurrence.

Various interventional strategies for peripapillary CNVM have been utilized with modest to moderate results for visual improvement. In studies using thermal laser alone, vision remained stable or improved in 75%–80% of eyes after treatment [1, 5–8]. However, MPS data for peripapillary CNVM demonstrated only a slight visual benefit with laser treatment. Only 50% of treated eyes retained 20/40 or better vision versus 44% in the untreated group in the study. Additionally, 14% of treated eyes versus 26% of untreated eyes lost 6 or more lines of vision [9]. Browning and Fraser [1] likewise reported modest visual improvement (7.8%) and increased worsening of vision (24.5%) in comparison, along with 67.5% of eyes as stable, after thermal laser treatment alone. Visual improvement was defined as a gain of greater than or equal to 3 lines, worse vision as a loss of 3 lines, and stability as either a loss or a gain of 2 lines. PDT data presented by Rosenblatt et al. [10] had an average of 2 lines of visual gain, while Bernstein and Horn [11] stated that all patients had visual gain. Studies involving surgical intervention for OHS reported 57.5% (23/40) of patients having 2 or more lines of visual gain [9].

Bevacizumab has been used alone to treat peripapillary CNVM [14, 15]. Figueroa et al. [14] treated 6 eyes with bevacizumab, including 2 after surgery and 1 eye with previous PDT treatment. Five eyes showed regression of the CNVM after 3 injections, while 1 eye required 6 injections. The reported visual outcomes were positive with an average of 4 lines increase in vision. Hoeh et al. [15] likewise demonstrated inactivation of CNVM lesions with an average of 3.5 injections (range 1–8). However, the follow-up after the last injection was rather brief, thus limiting available data regarding long-term visual improvement or stability.

In this study, using a combination of bevacizumab and thermal laser, 5/5 eyes (100%) had visual improvement with 4/5 eyes (80%) gaining 3 or more lines of vision. One patient, whose eye initially improved by 2 lines, ultimately resulted in 1 line of visual gain due to both eyes worsening from cataracts unrelated to the retina during the follow-up period. Four out of 5 patients were phakic, and 3 of the 4 eyes had moderately significant cataracts. Interestingly, the final vision obtained for these patients was the same in each eye. Thus, it is possible that cataract removal may lead to further visual improvements.

In each case at presentation, the CNVM was surrounded with subretinal hemorrhage, exudates, and subfoveal fluid resulting in decreased vision. After a series of 3 intravitreal bevacizumab injections, on average, the hemorrhage, subfoveal fluid, and exudates were resolved and the entire lesion was well-defined clinically and on FA. This allowed for direct and precise treatment of the CNVM with thermal laser. Since the total area of the CNVM was smaller after the bevacizumab injections, the total area of retina requiring treatment was also much smaller compared to the initial presentation, limiting excess thermal damage to the neurosensory retina. The use of bevacizumab or laser photocoagulation alone has also been associated with recurrence of subretinal fluid. Therefore, a single injection of bevacizumab was given following thermal laser to limit potential upregulation of VEGF and regrowth of CNVM due to photocoagulation.

Recurrence is a significant measure of therapeutic success and patients' improvement of quality of life. Previous studies using thermal laser treatment alone reported recurrence rates from 19% to 28% [1, 5–8]. Likewise, for treatments with intravitreal bevacizumab alone, Davis et al. [18] reported hemorrhage recurrence in nearly 30% of eyes during an average of 8.3 months follow-up following an average of 5.6 total injections. Subretinal fluid for 15% of eyes never resolved. High recurrence rates have also been reported with the use of other treatment modalities. Rosenblatt et al. [10] treated 7 patients with PDT and 5/7 (71.4%) required retreatment. Bernstein and Horn [11] likewise treated 7 patients with PDT and 2/7 patients (28.6%) required retreatment with PDT despite 4/7 (57.1%) of these patients having been previously treated with other modalities including intravitreal triamcinolone, pegaptanib, or ranibizumab. In studies involving surgical removal of CNVM associated with OHS, the recurrence rate was 22.5% (9/40 patients).

In this study, we report no recurrences with our treatment regimen. It is likely that the bevacizumab not only caused the involution of the CNVM but also aided in the reabsorption of the hemorrhage and exudates via inhibition of vascular endothelial growth factor (VEGF). In turn, this allowed for a more precise and thorough laser treatment of the entire lesion with a 200-micron temporal border. Moreover, recurrence has been shown to occur at lesion edges within the first 6 weeks to one year after treatment with thermal laser alone [19]. Recurrence or persistence of CNVM, or development of new CNVM and further visual deterioration after adequate thermal laser surgery, is usually a result of disease process and is not a complication. The bevacizumab injection given shortly after the thermal laser treatment likely inhibited this recurrence mechanism by mitigating potential upregulation of VEGF associated with laser treatment. Recurrence was absent for all patients in this study well beyond one year of follow-up, with an average follow-up of 24 months. While the results of this series are clearly positive, the study is limited by its retrospective design, small sample size, and the use of non-ETDRS vision. Further investigation is warranted with greater patient numbers and updated study design.

Bevacizumab and thermal laser combination therapy appears to be a promising treatment option for peripapillary CNVM. Initial treatment with bevacizumab may resolve hemorrhage-, edema-, and exudate-associated CNVM, reducing the total area of CNVM and retina requiring thermal laser. This strategy may also limit the total number of bevacizumab injections required, minimizing adverse consequences, while achieving increased visual acuity that remains stable over extended periods of time without recurrence. Together, this points to bevacizumab and thermal laser combination therapy as a favorable treatment strategy for improving patient visual function and decreasing CMT, while limiting the potential for recurrence and adverse effects.

## Conflicts of Interest

The authors declare that there is no conflict of interest regarding the publication of this paper.

## Figures and Tables

**Table 1:** Summary of data of patients treated with Avastin followed by thermal laser and Avastin for peripapillary CNVM.

Patient	Baseline		Number of injections prior to combo therapy	Final		Number of months of follow-up after combo therapy
	VA	CRT		VA	CRT	
1	20/40	328	2	20/20	186	26
2	20/80	347	3	20/40	152	28
3	20/40	367	3	20/20	145	21
4	20/50	^∗^	2	20/40	130	23
5	20/70	^∗^	4	20/30	181	22

VA, visual acuity; CRT, central retinal thickness; ^∗^information not available.
